# The complete mitochondrial genome of *Cuspidaria undata* (Bivalvia, Anomalodesmata, Cuspidariidae)

**DOI:** 10.1080/23802359.2023.2167478

**Published:** 2023-01-26

**Authors:** Yating Bao, Meiling Ge, Yaoyao Zhao, Zhou Zheng, Qinzeng Xu

**Affiliations:** a Key Laboratory of Marine Eco-Environmental Science and Technology, First Institute of Oceanography, MNR, Qingdao, China; b Department of Specialty Medicine, School of Basic Medicine, Qingdao University, Qingdao, China; c Laboratory for Marine Drugs and Bioproducts, Qingdao National Laboratory for Marine Science and Technology, Qingdao, China

**Keywords:** *Cuspidaria undata*, mitochondrial genome, phylogeny, Cuspidariidae

## Abstract

The mitochondrial genome of *Cuspidaria undata* (Verrill, 1884) was sequenced in full using Illumina HiSeq 2500. The circular mitochondrial DNA (mtDNA) was 16,266 bp in size, encoded 37 genes, and contained 13 protein-coding genes (PCGs), 2 rRNAs and 22 tRNAs. The gene order of the 13 PCGs in this species exhibited extensive rearrangement and differences in comparison to other Cuspidariidae, indicating that gene order is not conserved within this family. Phylogenetic analysis based on 13 PCGs and 2 rRNAs recovered a monophyletic Cuspidariidae.

The family Cuspidariidae Dall, 1886. belongs to the class Bivalvia, within the superorder Anomalodesmata (Worms 2020), and includes 19 genera and 254 species (Morton and Machado 2019). Cuspidariidae species are deep-sea predatory bivalves and are the most diversified group among the septibranch bivalves (Amano and Kurita 2020). Deep water mollusks have been sparsely studied and only one complete mitogenome of Cuspidariidae has previously been sequenced, one which belongs to the genus *Tropidomya* Dall & Smith, 1886 (Williams et al. 2017). In this study, the complete mitochondrial genome of *Cuspidaria undata* (Verrill, 1884) was sequenced to understand the genomic structure and phylogenetic relationships within this family (Figure 1).

A specimen of *C. undata* was collected from the Southern Indian Ocean (16.95110S, 89.66835E) during the China Ocean 52th voyage. The sample (Accession No. FIO2018015207; Contact person: Zhou Zheng, zhengzhou@fio.org.cn) was stored at −80 °C at the Key Laboratory of Marine Eco-Environmental Science and Technology, First Institute of Oceanography, Ministry of Natural Resources. Total DNA was extracted from muscle tissue using a DNeasy Blood & Tissue DNA kit (QIAGEN) and thereafter sequenced using the Illumina HiSeq 2500 Sequencing Platform (Beijing, China). The mitochondrial genome was assembled using NOVOplasty (Dierckxsens et al. 2017) and annotated using the MITOS Web Server (Bernt et al. 2013). Bandage (Wick et al. 2015) was used to verify the circular structure of the mitochondrial genome. The complete mitogenome of *C. undata* was submitted to GenBank, registration number ON360998.1.

The mitochondrial genome of *C. undata* was 16,266 bp in length, and contained 13 PCGs, 2 rRNAs and 22 tRNAs. The mtDNA of *C. undata* composition was 26.1% A, 10.4% C, 21.4% G, and 42.1% T. The percentage of A + T with the *C. undata* mtDNA was 68.2%. The 22 tRNA-coding genes ranged in size from 60 bp through to 74 bp. The gene order of the 13 PCGs was *COX1*, *NAD6*, *COB*, *NAD4L*, *COX2*, *ATP6*, *NAD2*, *NAD4*, *ATP8*, *NAD1*, *NAD5*, *NAD3* and *COX3*, which exhibited extensive rearrangement in comparison to the gene order previously published *Tropidomya abbreviata* (Forbes, 1843), also within Cuspidariidae (Williams et al. 2017), indicating that the mitochondrial gene order of this family is not conservative. In addition, we made a table describing the characteristics of *C. undata* mitochondrial genome (Table 1), and drew the mitochondrial genome map of *C. undata* (Figure 2) using OrganellarGenomeDRAW (OGDRAW) version 1.3.1 (Greiner et al. 2019).

**Figure 1.**
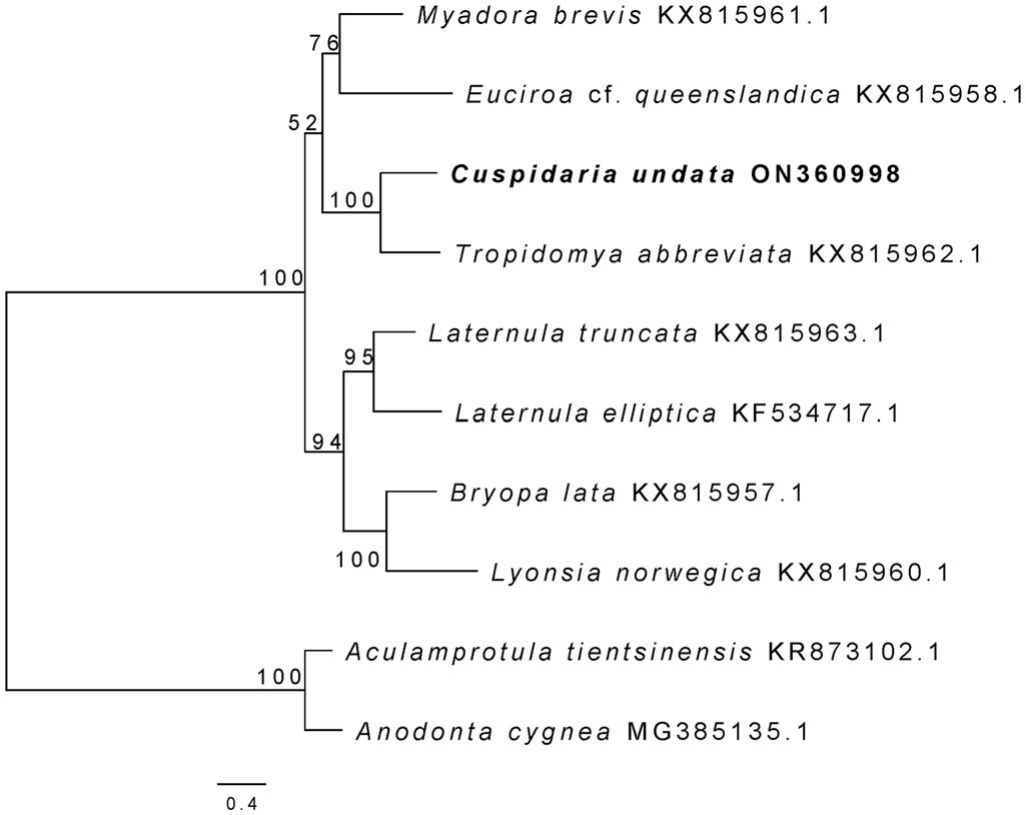
The maximum likelihood tree of 10 bivalves based on 13 PCGs and 2 rRNAs. Number at branch represents bootstrap probability. The following sequences were used: KX815961.1 (Williams et al. 2017), KX815958.1 (Williams et al. 2017), KX815962.1 (Williams et al. 2017), KX815963.1 (Williams et al. 2017), KF534717.1 (Park and Ahn 2015), KX815957.1 (Williams et al. 2017), KX815960.1 (Williams et al. 2017), KR873102.1 (Wu et al. 2016), MG385135.1 (Soroka and Burzyński 2017).

**Figure 2.**
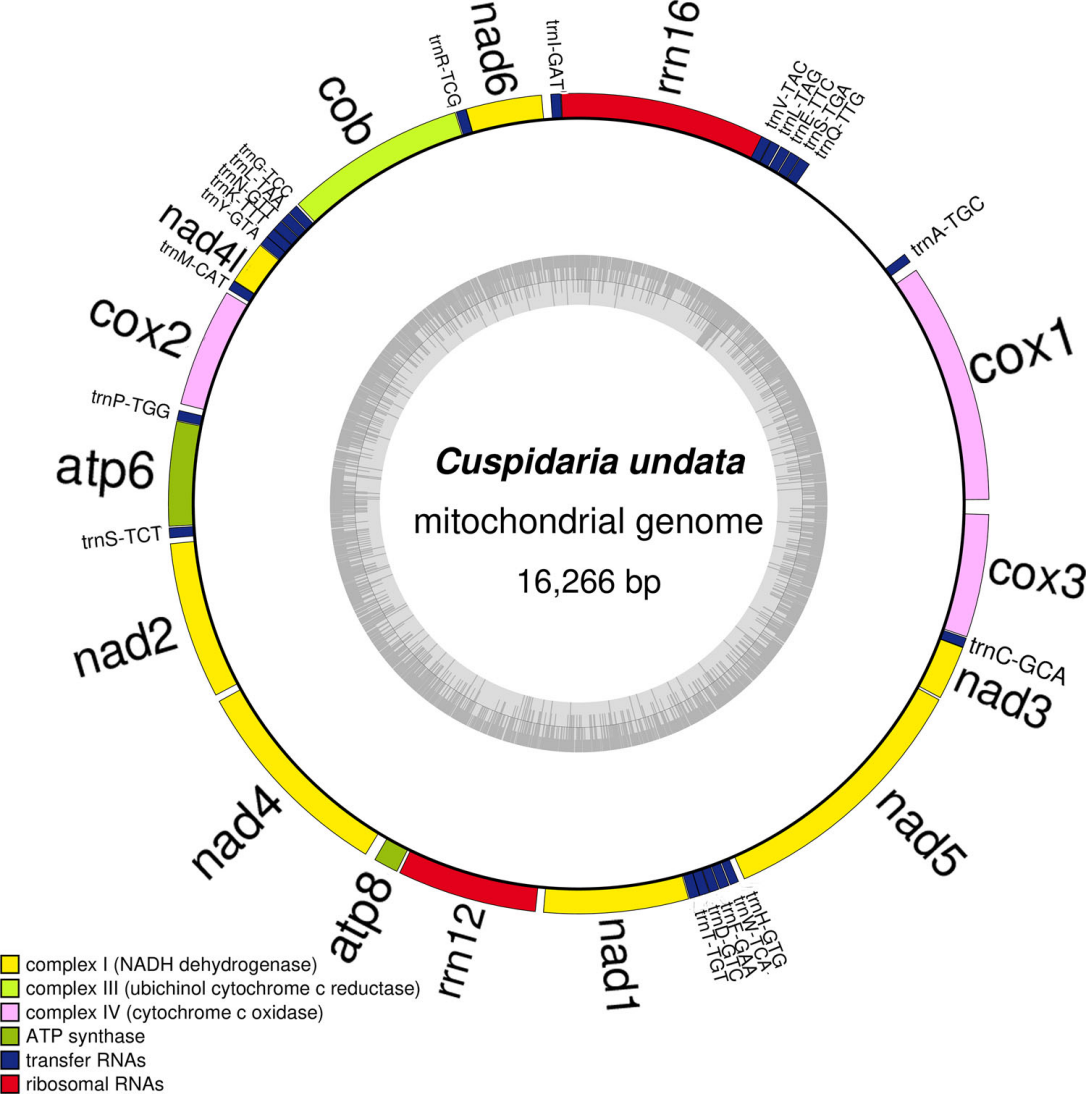
Map of the mitochondrial genome of *C. undata*. The mitochondrial genome of *C. undata* was 16,266 bp in length, and contained 13 PCGs, 2 rRNAs and 22 tRNAs, marked in different colors in the map.

**Table 1.** Mitochondrial genome of *C. undata* with gene order, positions, lengths, nucleic acid composition, coding strand, start and stop codons, anticodons and amino acid size.

Gene	Position		Length (bp)	Nucleic acid		Coding strand	Codon		Anticodons	Amino acid size
	Start	End		AT (%)	GC (%)		Start	Stop		
cox1	25	1569	1545	63.49	36.51	+	GTT	TAA		514aa
trnA	1629	1693	65	70.77	29.23	+			TGC	
trnQ	2519	2592	74	68.92	31.08	+			TTG	
trnS	2594	2663	70	61.43	38.57	+			TGA	
trnE	2667	2734	68	75.00	25.00	+			TTC	
trnL	2741	2804	64	65.62	34.38	+			TAG	
trnV	2805	2872	68	83.82	16.18	+			TAC	
rrn16	2855	4178	1324	73.04	26.96	+				
trnI	4179	4245	67	64.18	35.82	+			GAT	
nad6	4300	4788	489	69.53	30.47	+	ATG	TAG		162aa
trnR	4787	4853	67	70.15	29.85	+			TCG	
cob	4857	6017	1611	68.48	31.52	+	ATA	TAA		386aa
trnG	6029	6094	66	66.67	33.33	+			TCC	
trnL	6097	6164	68	67.66	32.84	+			TAA	
trnN	6166	6234	69	75.36	24.64	+			GTT	
trnK	6235	6300	66	57.58	42.42	+			TTT	
trnY	6298	6363	66	60.61	39.39	+			GTA	
nad4l	6366	6647	282	67.73	32.27	+	ATG	TAA		93aa
trnM	6648	6712	65	63.08	36.92	+			CAT	
cox2	6734	7498	765	63.40	36.60	+	ATG	TAG		254aa
trnP	7538	7604	67	76.12	23.88	+			TGG	
atp6	7607	8278	672	67.71	32.29	+	ATG	TAA		223aa
trnS	8285	8352	68	64.71	35.29	+			TCT	
nad2	8385	9392	1008	68.06	31.94	+	ATT	TAA		335aa
nad4	9433	10788	1356	68.44	31.56	+	ATG	TAA		451aa
atp8	10852	11013	162	72.84	27.16	+	ATG	TAA		53aa
rrn12	11029	11927	899	69.86	30.14	+				
nad1	11975	12904	930	69.14	30.86	+	ATA	TAG		309aa
trnT	12908	12975	68	73.53	26.47	+			TGT	
trnD	12974	13040	67	79.10	20.90	+			GTC	
trnF	13041	13104	64	67.19	32.81	+			GAA	
trnW	13107	13173	67	68.66	31.34	+			TCA	
trnH	13178	13237	60	75.00	25.00	+			GTG	
nad5	13274	14977	1704	69.07	30.93	+	ATT	TAA		567aa
nad3	14989	15339	351	69.52	30.48	+	GTG	TAG		116aa
trnC	15339	15402	64	65.63	34.37	+			GCA	
cox3	15408	16199	792	63.38	36.62	+	ATG	TAG		263aa

The maximum likelihood (ML) phylogenetic tree of *C. undata* was constructed based on the 13 PCGs and 2 rRNAs, containing seven other Anomalodesmata species and two Palaeoheterodonta species as outgroups. Nucleotide sequences from each mitogene were aligned using MAFFT v7.313 (Katoh et al. 2002) and then trimmed with Gblocks 0.9 b (Talavera and Castresana 2007). All of the aligned gene sets were concatenated using SequenceMatrix v1.7.8 (Vaidya et al. 2011). The best fitting nucleotide-substitution model of each partition was evaluated using PartitionFinder2 (Lanfear et al. 2017). ML analysis was analyzed using IQ-TREE v. 1.6.8 (Nguyen et al. 2015) with 1,000 bootstrap replicates. The phylogenetic position of *C. undata* (ON360998.1) was sister to *T. abbreviata* and the family Cuspidariodea recovered a monophyletic clade.

## Data Availability

The genome sequence data that support the findings of this study are openly available in GenBank of NCBI (https://www.ncbi.nlm.nih.gov/nuccore/ON360998) (GeneBank Number: ON360998.1). The associated BioProject, SRA, and Bio-Sample numbers are PRJNA848421, SRR19647706, and SAMN29005581, respectively.
